# Supplementary material for: Robust broad spectral photodetection (UV-NIR) and ultra high responsivity investigated in nanosheets and nanowires of Bi2Te3 under harsh nano-milling conditions
Source: Sci Rep. 2017 Dec 20;7:17911. doi: 10.1038/s41598-017-18166-4 (PMC5738343; doi:10.1038/s41598-017-18166-4)
Supplement: Supplementary file 1 — Supplementary Information [file 41598_2017_18166_MOESM1_ESM.pdf]

Robust broad spectral photodetection (UV-NIR) and ultra high responsivity investigated in nanosheets and nanowires of Bi_2Te_3 under harsh nano-milling conditions

Alka Sharma[1,2], A. K. Srivastava[1,2], T. D. Senguttuvan[1,2] and Sudhir Husale[1,2]*

[1]Academy of Scientific and Innovative Research (AcSIR), National Physical Laboratory, Council of Scientific and Industrial Research, Dr. K. S Krishnan Road, New Delhi-110012, India.

[2] National Physical Laboratory, Council of Scientific and Industrial Research, Dr. K. S Krishnan Road, New Delhi-110012, India.

*E-mail: husalesc@nplindia.org

Supplementary Information contents:

1. Rise and Decay time fit
2. Optoelectronic characterization of Bi_2Te_3 nanosheets
3. Optoelectronic properties of Bi_2Te_3 nanowires (NW)
4. Robustness and enhancements in photoconduction measurements
5. Photoconductivity of (visible) of NW1 device
6. Photoconductivity of (NIR) of NW2 device
7. Photoconductivity of (NIR) of Fab_NW1, Fab_NW2 and Fab_NW3 devices
8. Photoconducting gain of the device (Fab_NW3)

1. Rise and Decay time fit

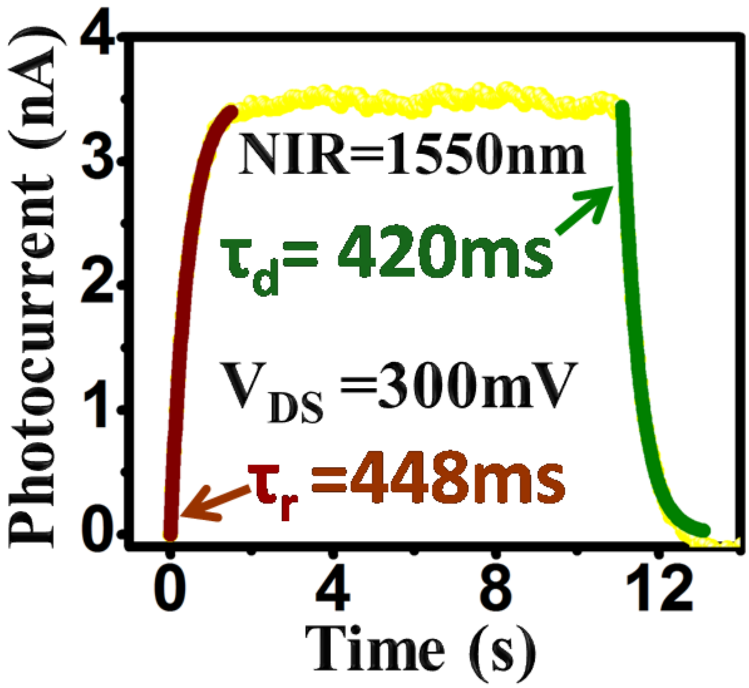

Figure S1: Rise (red) and decay (green) curve fitting.

2. Optoelectronic characterization of Bi_2Te_3 nanosheets

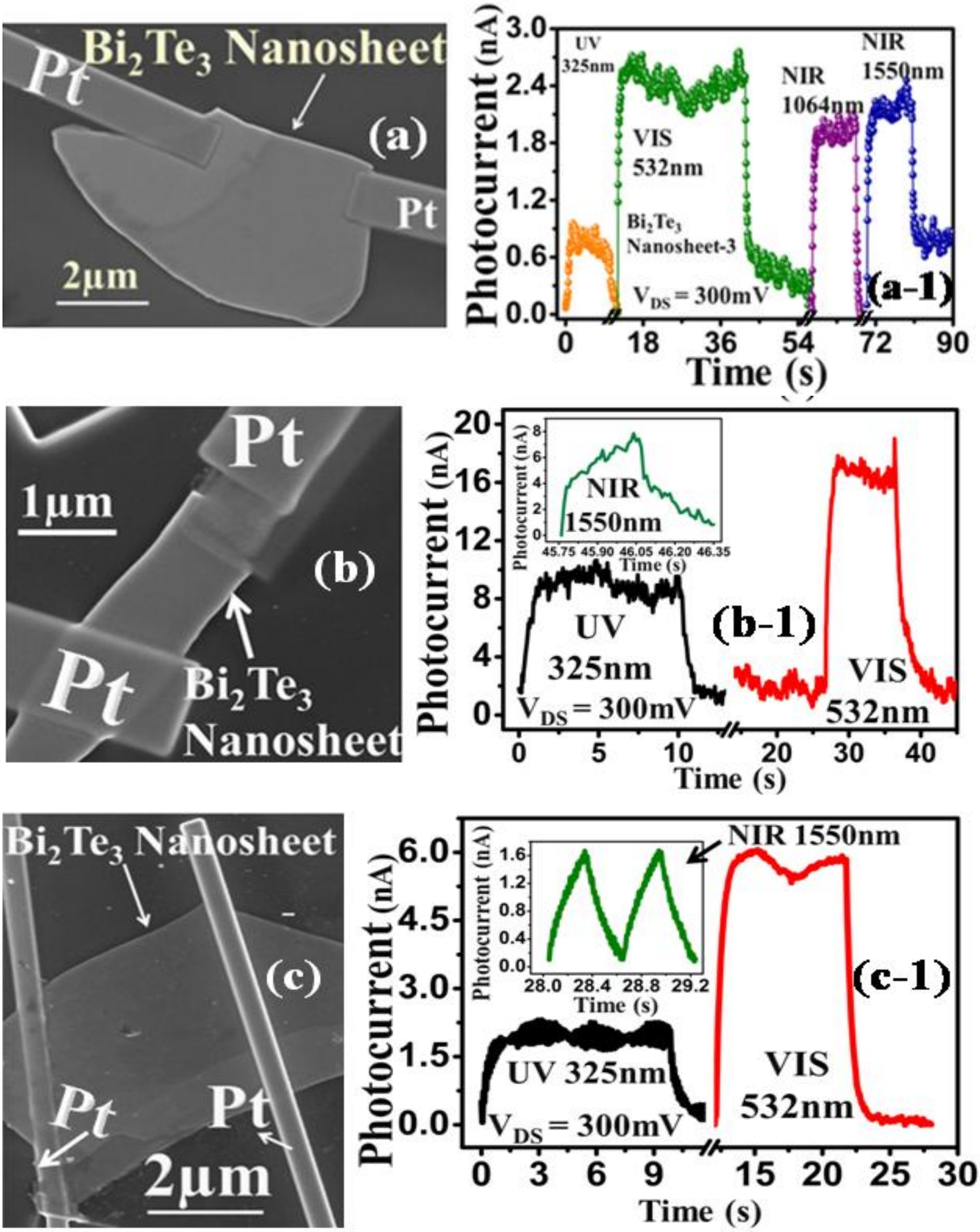

Figure S2: Photoconductivity measurements on three more nanosheet devices

3. Optoelectronic characterization of Bi_2Te_3 nanowire

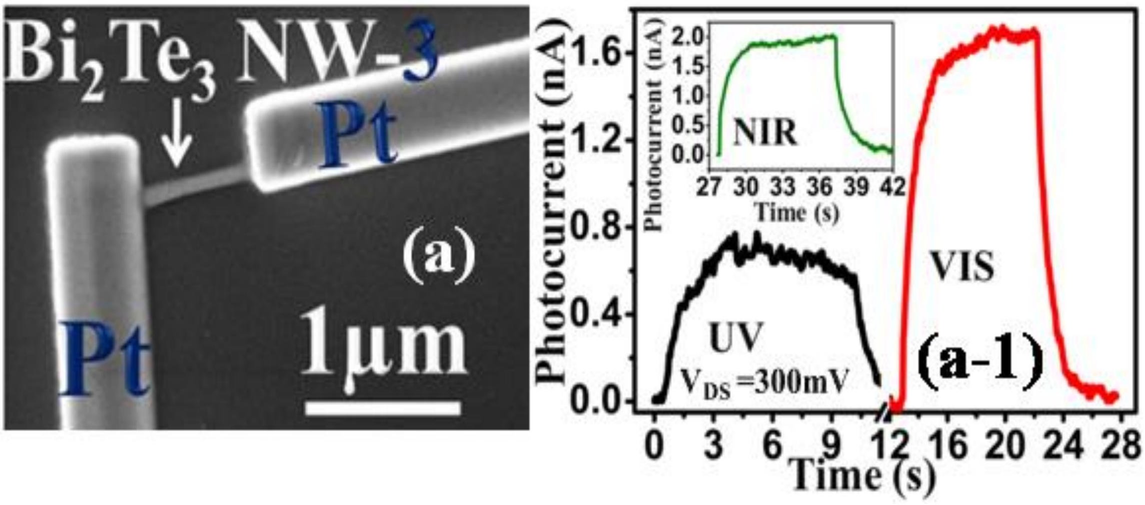

Figure S3: Photoconductivity measurements on one more fabricated nanowire device

4. Robustness and enhancements in photoconduction measurements

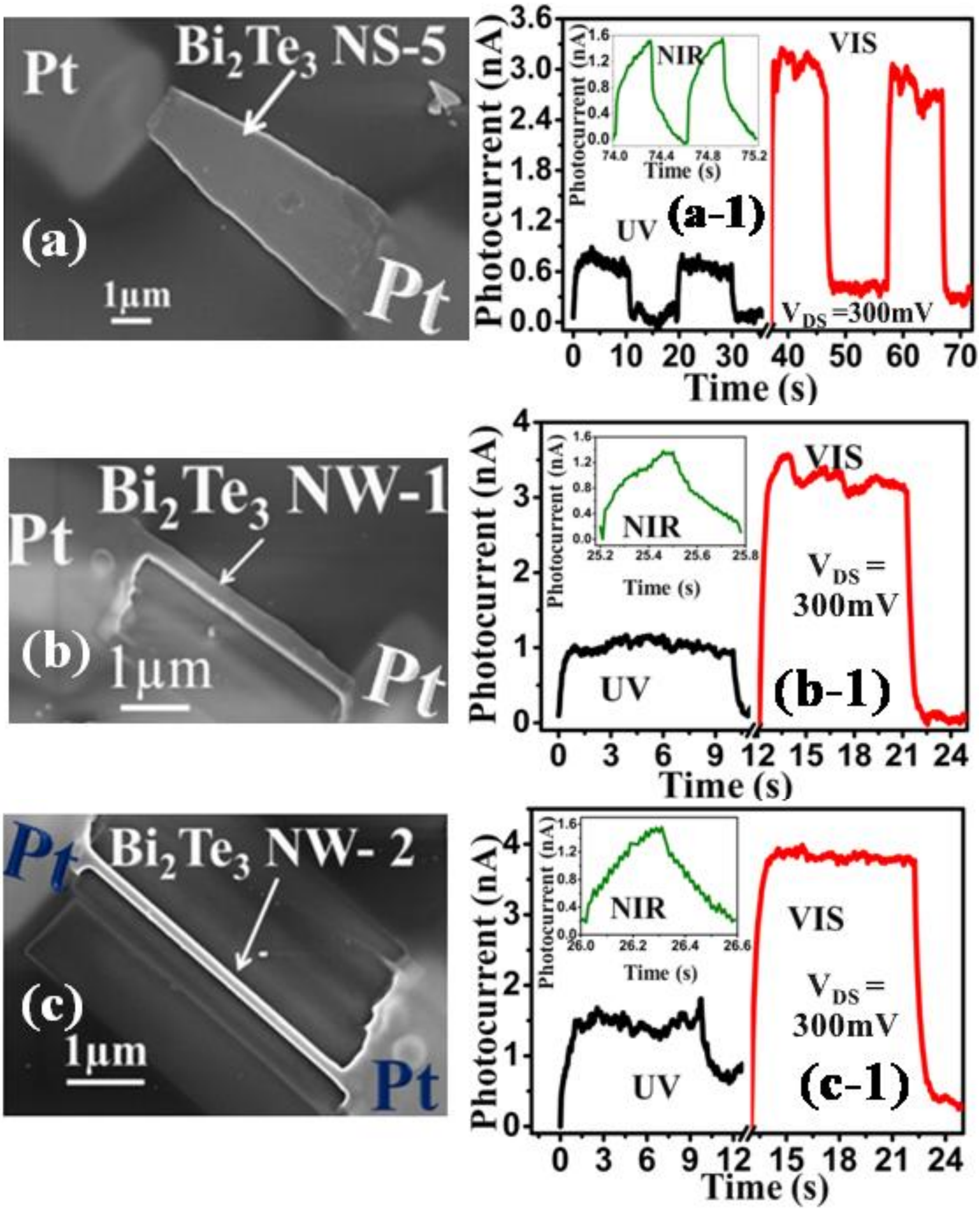

Figure S4: Photoconductivity measurements investigating robustness under harsh nano milling conditions

5. Photoconductivity of (visible) of NW1 device

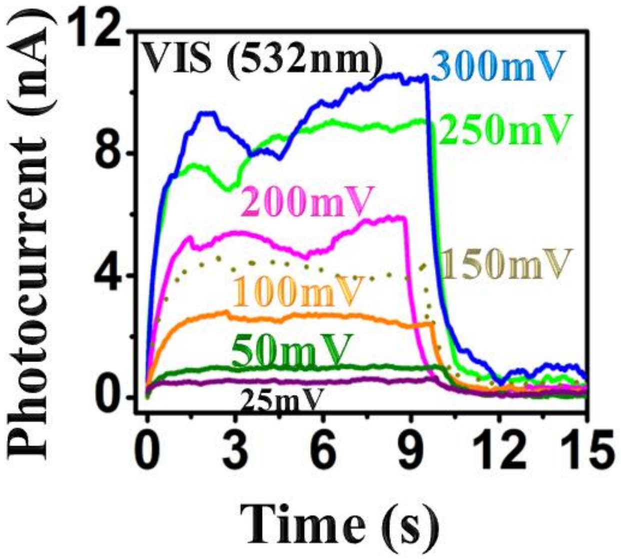

Figure S5: Bias voltage dependent photoconductivity measurements under illumination of visible light and device is NW1.

6. Photoconductivity of (NIR) of NW2 device

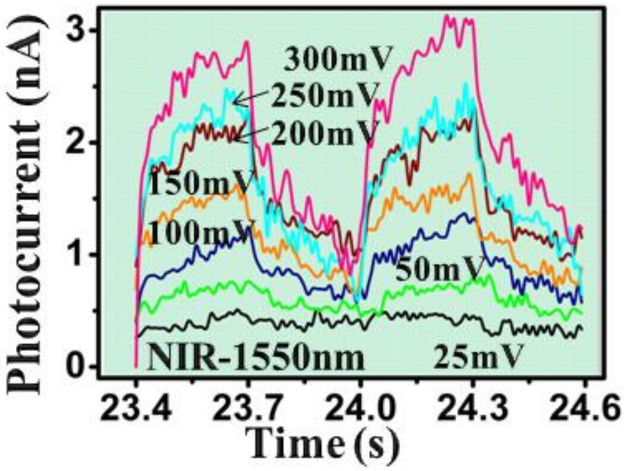

Figure S6: Bias voltage dependent photoconductivity measurements under illumination of NIR light and device is NW2.

7. Photoconductivity of (NIR) of Fab_NW1, Fab_NW2 and Fab_NW3 devices

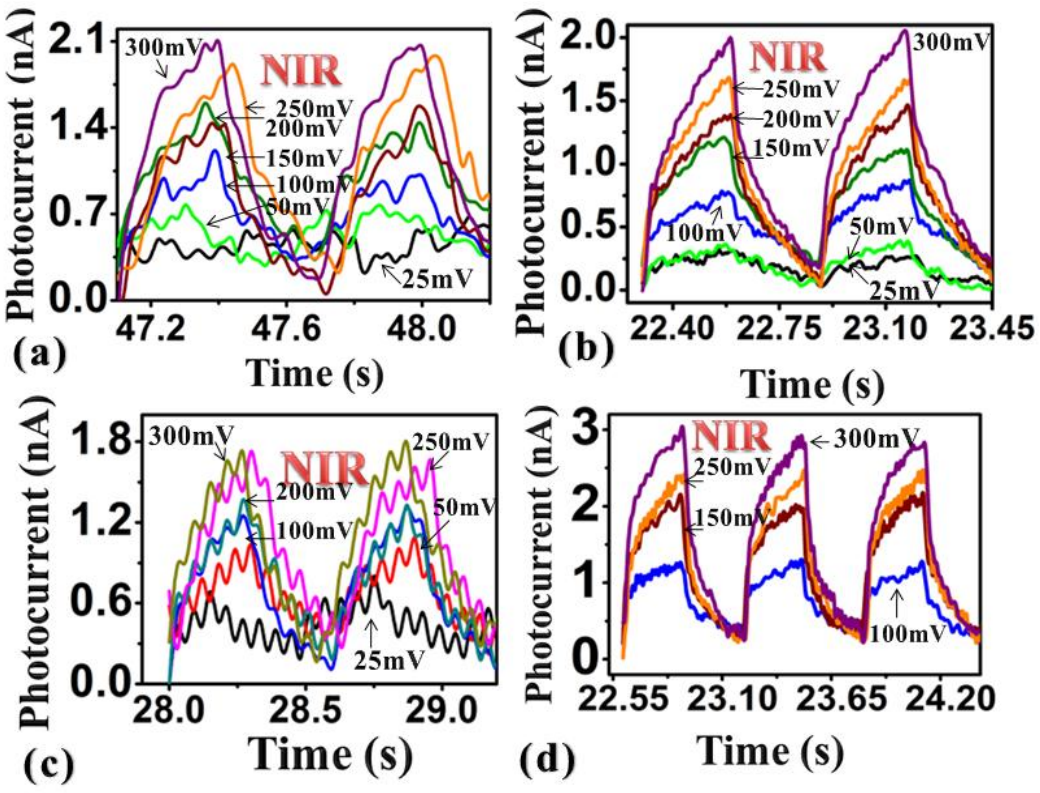

Figure S7: Bias voltage dependent photoconductivity measurements under illumination of NIR light and devices are Fab_ NW1, Fab_ NW2 and Fab_ NW3.

8. Photoconducting gain of the device (Fab_NW3)

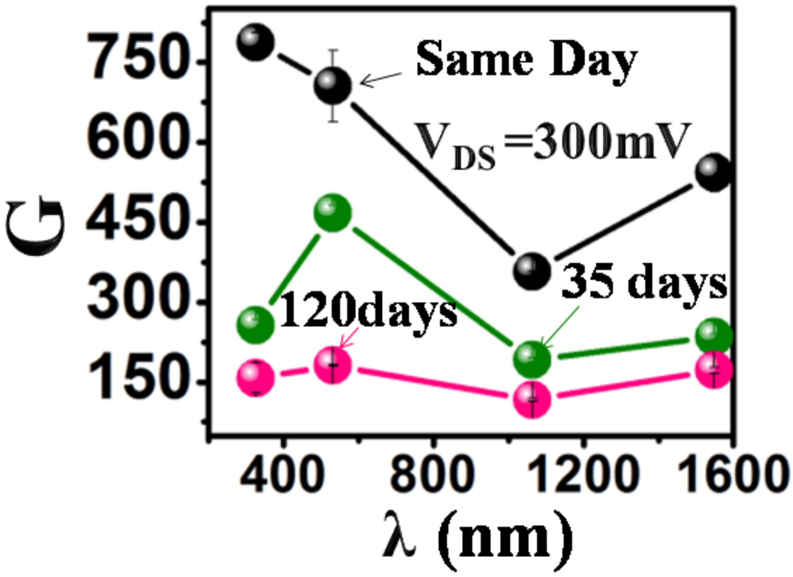

Figure S8: Photoconductive gain of the device Fab_NW3.
